# Identification of SARS‐CoV‐2 RNA in healthcare heating, ventilation, and air conditioning units

**DOI:** 10.1111/ina.12898

**Published:** 2021-06-29

**Authors:** Patrick F. Horve, Leslie G. Dietz, Mark Fretz, David A. Constant, Andrew Wilkes, John M. Townes, Robert G. Martindale, William B. Messer, Kevin G. Van Den Wymelenberg

**Affiliations:** ^1^ Biology and the Built Environment Center University of Oregon Eugene OR USA; ^2^ Institute for Health in the Built Environment University of Oregon Portland OR USA; ^3^ Department of Molecular Microbiology and Immunology Oregon Health and Science University Portland OR USA; ^4^ Healthcare Facilities Oregon Health and Science University Portland OR USA; ^5^ Division of Infectious Diseases Department of Medicine School of Medicine Oregon Health and Science University Portland OR USA; ^6^ Division of Gastrointestinal and General Surgery School of Medicine Oregon Health and Science University Portland OR USA

**Keywords:** aerosols, COVID‐19, filtration, healthcare, HVAC, MERV, SARS‐CoV‐2

## Abstract

Evidence continues to grow supporting the aerosol transmission of severe acute respiratory syndrome coronavirus 2 (SARS‐CoV‐2). To assess the potential role of heating, ventilation, and air conditioning (HVAC) systems in airborne viral transmission, this study sought to determine the viral presence, if any, on air handling units in a healthcare setting where coronavirus disease 2019 (COVID‐19) patients were being treated. The presence of SARS‐CoV‐2 RNA was detected in approximately 25% of samples taken from ten different locations in multiple air handlers. While samples were not evaluated for viral infectivity, the presence of viral RNA in air handlers raises the possibility that viral particles can enter and travel within the air handling system of a hospital, from room return air through high‐efficiency MERV‐15 filters and into supply air ducts. Although no known transmission events were determined to be associated with these specimens, the findings suggest the potential for HVAC systems to facilitate transfer of virions to locations remote from areas where infected persons reside. These results are important within and outside of healthcare settings and may present necessary guidance for building operators of facilities that are not equipped with high‐efficiency filtration. Furthermore, the identification of SARS‐CoV‐2 in HVAC components indicates the potential utility as an indoor environmental surveillance location.

**Practical Implications**

- More work is needed to further evaluate the risk of SARS‐CoV‐2 transmission via HVAC systems and to verify effectiveness of building operations mitigation strategies for the protection of building occupants.
- The results of this study suggest that building occupants, homeowners, and buildings and facility managers should review their air filtration and ventilation practices accordingly.
- Evaluating building HVAC operational practices and equipment and implementing enhancements could reduce future built environment transmission risks as more buildings reopen or increase in occupancy.
- This study suggests the potential for AHUs to serve as a location to conduct environmental viral surveillance to guide building operations, human behavior, and other mitigation actions according to the relevant risks identified.

## 1 INTRODUCTION

Since its emergence in late 2019, SARS‐CoV‐2 has spread across the globe and led to the deaths of over 1 400 000 individuals.^1^ Although most health organizations and agencies describe the main mechanism of transmission as respiratory droplet transmission by symptomatic or asymptomatic persons,^2^, ^3^, ^4^, ^5^, ^6^ there is significant growing evidence that aerosol transmission plays a major role in the spread of SARS‐CoV‐2 indoors and of COVID‐19 transmission.^7^, ^8^, ^9^, ^10^, ^11^, ^12^, ^13^, ^14^, ^15^ Additionally, recent studies suggest that air movement patterns indoors induced through heating, ventilation, and air conditioning (HVAC) systems may contribute to transmission events.^16^, ^17^

Aerosolized SARS‐CoV‐2 RNA has been previously detected in the air of hospital rooms with symptomatic COVID‐19 patients,^18^, ^19^, ^20^, ^21^ suggesting the possibility that SARS‐CoV‐2 viral RNA (and potentially virus) have the capacity to enter into building HVAC systems via evacuated room air after a shedding or aerosolization event from infected individuals. Although hospitals contain higher levels of mechanical filtration and room air exchange than almost all other buildings, which are important strategies to help prevent the transmission of disease, a growing body of evidence suggests that these precautions may not be adequate to completely eliminate SARS‐CoV‐2^10^, ^22^, ^23^ in filtered air. Furthermore, the identification of viable SARS‐CoV‐2 in the air of COVID positive patient rooms further underlies the need to better understand aerosol dynamics of SARS‐Cov‐2 spread indoors.^12^ Studies have shown the persistence of SARS‐CoV‐2 to be hours in the air and on surfaces.^24^ Efforts to limit the transmission and continued spread of SARS‐CoV‐2 have mainly focused on social (spatial) distancing, increased cleaning regimens, mandated face coverings, and increased surveillance.^24^, ^25^ However, as more indoor spaces begin to reopen and increase in occupant density, more individuals will occupy shared air spaces serviced by HVAC units for extended periods of time. Without an improved understanding of the risk posed from recirculated air in these indoor environments, there are potential gaps in the prevention and mitigation plans aimed to reduce SARS‐CoV‐2 transmission and thereby the number of COVID‐19 cases.

In the past, ventilation has played a key role in the transmission of infectious disease.^17^, ^26^, ^27^, ^28^ Similarly, there are several reported hospital‐associated SARS‐CoV‐2 infections and outbreaks during the COVID‐19 pandemic.^29^, ^30^ With the demonstration that ventilation systems may contribute to occupant transmission events, HVAC and ventilation guidelines and recommendations have been modified.^31^, ^32^, ^33^ Here, we present data demonstrating the presence of SARS‐CoV‐2 RNA at several locations along mechanical ventilation air return and supply pathways, including multiple locations in air handling units (AHUs).

## 2 MATERIALS AND METHODS

### 2.1 Sample collection

Samples were collected from Oregon Health and Science University (OHSU) hospital in Portland, Oregon, USA on four sampling days in May and June 2020. Environmental sampling does not require Institutional Review Board (IRB) approval; however, the project was reviewed by the OHSU IRB and an IRB Exemption was granted for this work. This work was reviewed by the OHSU Institutional Biosafety Committee and approved under PROTO202000016.

**FIGURE 1:**
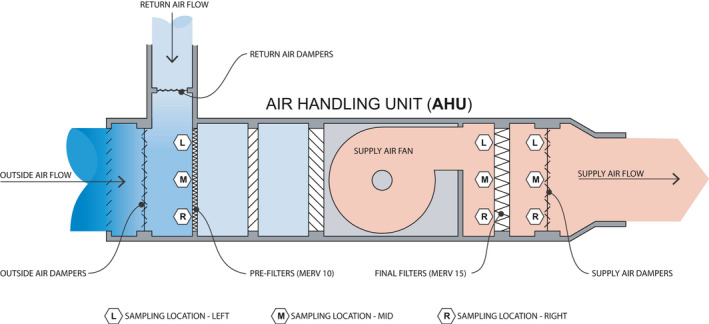
AHU sectional diagram illustrating the path of airflow, mixing of recirculated return air with outdoor air fraction and locations of swab sampling

During May and June 2020, samples were collected from three separate AHUs (Figure 1) serving wards with COVID‐19 patients. COVID‐19 positive patients were determined through either an initial rapid SARS‐CoV‐2 antigen test followed by a quantitative reverse‐transcription polymerase chain reaction (RT‐PCR) diagnostic test or simply a RT‐PCR diagnostic test. The AHUs have a minimum outside air percentage of 40% by design. The percentage of outside air was determined using return air temperature, supply air temperature, and mixing air temperature (Table 1). COVID‐19 positive patients were housed in ward 6A (emergency department), 7C (MICU), 12C (Labor and Delivery), and 14C (Internal Medicine Inpatient) that were serviced by the three sampled AHUs. These AHUs were selected because they handled air from areas with confirmed COVID‐19 positive patients. When space allowed, COVID‐19 positive patients were first placed in airborne infection isolation (AII) rooms that vent directly to the outside. Additional patients were cohorted on 6A, 7C, 12C, and 14C that employ mixing ventilation with air supply diffusers and return grills located on the ceiling in patient rooms. Within each AHU, three areas along the path of airflow were sampled, including the pre‐filters, final filters, and supply air dampers (Figure 1). A summary of the most recent maintenance data can be found in Table 2. The pre‐filters are rated at MERV10 and final filters are rated at MERV15, both in excess of minimum code requirements.^34^ MERV10 and MERV15 filters carry a capture efficiency of 50% and 90% respectively for particles ranging from 1–3 microns in size. Based upon engineering calculations and equipment documentation available, the HVAC system is capable of cycling air from the ward, to the AHU, and back to the ward in a time between 90 sec and 5 min, depending on travel distance to room location. All filters used in the AHU filter blocks are manufactured by Camfil (Models 3V and 5V). Ultraviolet light disinfection was not utilized in the AHUs sampled during this study. Samples were collected using Puritan PurFlock Ultra swabs (catalog #25‐3606‐U) and swabs were taken from the left, middle, and right sides of each filter block along the path of airflow. Swabs were individually processed. Samples were only collected from the intake side of the filters. Swabs were pre‐moistened using viral transport media (RMBIO, catalog #VTM‐CHT). Swabbing occurred for 20 sec on an area approximately 20 × 30 cm at each location and swabs were immediately placed into 15 ml conical tubes (Cole‐Parmer, catalog #UX‐06336‐89) containing 1.5 ml viral transport media and stored on ice for transport to a BSL‐2 laboratory with enhanced precautions (BSL2+) lab for processing, which typically occurred within 2 h after collection. Samples were collected by the same researcher each sampling time, and the researcher did not demonstrate any symptoms of COVID‐19 and tested negative by qRT‐PCR.

**TABLE 1:** Sampling dates and calculated outside air percentage in each Air Handling Unit

Air Handler Unit (AHU)	Collection date	Outside air percentage (%)
1	15‐May‐20	86.5
	28‐May‐20	42.4
5	15‐May‐20	65.6
	9‐Jun‐20	93.3
	12‐Jun‐20	95
10	28‐May‐20	12.8
	12‐Jun‐20	100

**TABLE 2:** Available information on the most recent filter maintenance for the sample Air Handling Units

	AHU #1	AHU #5	AHU #10
Prefilter change date	14 May 2018	19 May 2020	11 March 2020
Final filter change date	19 April 2017	25 March 2020	26 September 2017

### 2.2 Sample processing and molecular analysis

Samples were hand‐carried to a research laboratory at OHSU for initial processing. In a class 2 biosafety cabinet (BSC), conical tubes were vortexed briefly, allowed to settle for 5 min, and 200 µl of the supernatant was removed and combined with 600 µl of the lysis/preservative buffer (DNA/RNA Shield, Zymo Research). Samples were then transported by car to a BSL‐2 laboratory at the University of Oregon campus in Eugene, Oregon, USA. Total RNA was extracted from all samples using Zymo Quick‐DNA/RNA Viral MagBead kit (Zymo Research #R2141) and stored at −80°C until analysis. The abundance of SARS‐CoV‐2 in each sample was determined using quantitative reverse‐transcription polymerase chain reaction (qRT‐PCR) targeting a 157 bp segment of the SARS‐CoV‐2 spike glycoprotein gene.^3^ An artificial gene standard from Integrated DNA Technologies with known copy number was utilized to create a dilution series and standard curve for the determination of viral gene copies in each sample,^35^ with a limit of detection of 2.22 gene copies/ul. All qRT‐PCR reactions were run in triplicate. Potential contamination during initial processing was assessed through the use of passive air settling plates and reagent controls within the BSL2 lab. To accomplish this, passive air settling plates were placed in the BSC and on the outside lab bench for the duration of processing. Following the completion of specimen processing, the same swabs, viral transport media, and processing steps were performed in an identical fashion to environmental samples for the controls. Reagent controls were processed concurrently with environmental samples. All controls tested negative for the presence of SARS‐CoV‐2 RNA. All samples that tested negative for the presence of SARS‐CoV‐2 RNA were included in the analysis by treating them as non‐detection samples instead of true zero samples^36^, ^37^, ^38^ in order to avoid creating a censored dataset. The full dataset can be found in Table S1. The samples were considered to contain one‐half the assay's limit of detection in gene copies/ul (1.1 gene copies/ul).

## 3 RESULTS

In total, 56 samples from three different AHUs were collected; 25% (14/56) of samples contained detectable SARS‐CoV‐2 RNA (Table S1). The highest abundance sample (~49 gene copies/ul) was found on the pre‐filters, where outside air mixes with recirculated building air. Of the samples collected, 25% (5/20) of samples at the pre‐filters, 17.6% (3/17) of samples at the final filter, and 31.6% (6/19) of samples at the supply air dampers contained detectable SARS‐CoV‐2 RNA (Table 3). The least SARS‐CoV‐2 RNA was detected at the final filter and the most at the pre‐filters (Table 3). No correlation between outside air percentage and detected genome copies was observed (Figure S1).

**TABLE 3:** Summary statistics and percent of positive samples from each sampling location within all AHUs

	Pre‐filters		Final filters		Supply air dampers	
	Total number (n)	Number positive (%)	Total number (n)	Number positive (%)	Total number (n)	Number positive (%)
	20	5 (25)	17	3 (17.6)	19	6 (31.6)
Cumulative gene copies (x̅)	87.6 (4.38)		36.2 (2.13)		82.9 (4.37)	

Note

Data is presented in SARS‐CoV‐2 genome copies per microliter of reaction.

## 4 DISCUSSION

This investigation demonstrates the presence of SARS‐CoV‐2 RNA at multiple locations within mechanical AHUs, and more specifically, AHUs serving multiple floors of a hospital tower in which COVID‐19 patients were housed. Furthermore, SARS‐CoV‐2 RNA remained detectable 31.6% (6/19) of the time at the final sampling location (supply air damper), after the recirculated air had been mixed with fresh outside air, passed through the pre‐filter (MERV10) and final filter (MERV15) stages. This suggests that the filtration practices in place in some of the most highly filtered environments, such as healthcare, does not eliminate the passage of SARS‐CoV‐2 viral RNA, and potentially SARS‐CoV‐2 viral particles, through HVAC systems and potentially back into the supply air. The infectious potential of this viral genetic material is currently unknown. These data demonstrate the potential that air evacuated from building spaces containing infectious individuals may be recirculated and distributed to other building spaces through centralized HVAC systems while containing SARS‐CoV‐2 RNA (and possibly virus), even after the filtration process and the dilution from the addition of 70%–80% outside air (thus, only 20%–30% recirculated air, during the sampling period).

Although positive samples were not assessed for infectivity of SARS‐CoV‐2, the presented data support previous claims of aerosolized viral particles^10^, ^17^, ^19^, ^21^ that may be carried remotely from their source by indoor air currents produced by built environment HVAC systems. Despite the growing evidence of the potential airborne nature of SARS‐CoV‐2 and the potential for aerosolized particles to contribute to some transmission events,,^5^, ^10^, ^16^, ^17^, ^19^, ^21^, ^39^, ^40^ guidance by both the World Health Organization^41^ (WHO) and Centers for Disease Control^42^ (CDC)^43^ concerning the potential for viral spread through airborne routes of transmission was only recently updated to include guidance against potential aerosol transmission of SARS‐CoV‐2 indoors. The American Society of Heating Refrigerating and Air‐Conditioning Engineers (ASHRAE) Standard 170–2017 Ventilation for Healthcare Facilities guidelines requires MERV 7 minimum for filter bank one and MERV 14 minimum for filter bank two in healthcare settings such as those studied here.^34^ The building studied herein exceeds requirements for filtration efficiency at both filter bank locations.

Negative pressure rooms reduce air leakage from the room to the ward. AII rooms typically vent room air directly to the outside and are required to have a minimum of 12 ACH and 2 ACH of outside air. Placement of patients with known or suspected COVID‐19 in AII negative pressure rooms when available, as recommended by CDC, greatly reduces the risk of recirculation of virus particles within HVAC systems by limiting air leakage to the ward and exhausting air directly outside. However, most hospitals and outpatient clinics do not have sufficient numbers of negative pressure rooms to accommodate all patients with known or suspected COVID‐19. Furthermore, a substantial number of infected individuals that are asymptomatic or pre‐symptomatic can shed aerosolized viral particles to shared air spaces.^44^ This risk of transmission is likely even higher in non‐healthcare buildings that often do not have qRT‐PCR based human diagnostic screening practices, and typically have lower air exchange rates and less efficient filtration, and during extreme weather conditions when HVAC thermal system capacities cannot manage thermal comfort with higher outside air fractions.

Previous studies have demonstrated that SARS‐CoV‐2 can be found in aerosols and droplets ranging from 0.25 to 4 microns.^10^, ^11^ MERV15 filters have a capture efficiency of 85%–90% for particles ranging from 0.3 to 3 microns in size. It is possible that particles containing SARS‐CoV‐2 RNA passed through the filters or bypassed the filters and were impacted on the supply air dampers. Additionally, it has been previously observed that small gaps in between filters from suboptimal installation may allow for biological material to pass through filters in hospital HVAC systems.^1^, ^2^ However, we did not set out to experimentally determine how SARS‐CoV‐2 genetic material may have bypassed filtering steps in the HVAC system, and any assertions as to the mechanism of bypass would be nothing more than speculation. In experimentally generated aerosols, SARS‐CoV‐2 and other coronaviruses have been demonstrated to retain infectivity for between one and sixteen hours,^45^, ^46^, ^47^, ^48^ lending credence to the potential for aerosolized transmission to occur. Even more, SARS‐CoV‐2 has been demonstrated to be infectious in aerosols in rooms of SARS‐CoV‐2 positive patients,^12^ suggesting the potential risk of transmission through HVAC systems, and warranting further investigation.

There are steps that can be taken to limit the potential impact of airborne dissemination of viruses in the built environment, including careful donning and doffing of personal protective equipment,^24^ hand hygiene after hand contact with the environment, cleaning of contaminated surfaces, use of UV radiation,^49^ ensuring indoor relative humidity is between 40% and 60%,^46^ and providing indoor ventilation pathways that extract contaminated air with minimal in‐room recirculation (such as displacement ventilation). Use of the highest possible efficiency filters for the building type and HVAC system design,^5^ and increasing the fraction of outside air introduced into indoor environments^5^ when possible are additional strategies that could mitigate transmission of virus through ventilation systems.

There were several limitations to this study. First, samples were not evaluated for the presence of viable SARS‐CoV‐2 virus. Second, to prevent disruption of hospital operations, routine sampling of all AHUs was not possible, limiting temporal analysis. Third, the sample point of a filter or damper can only be representative of that sampling area and the porous nature of the filters may inhibit efficient specimen recovery. Additionally, surface swabs are inherently not perfectly efficient and differential from one sampling time to another. Lastly, this is a focused examination of one specific exposure factor and does not address several others (exposure routes, sampling conditions, viability) and should therefore only serve as part of the equation in understanding the overall exposure risk of SARS‐CoV‐2.

Given that surface swabs collect a time‐integrated signal that may include RNA from as long as 17 days^50^ before the time of sampling, meaningful variation in viral load per sampling day was not expected. However, in order to explore this possibility and better characterize the viral abundances collected, hospital COVID‐19 census data were collected retrospectively for the sampling days. Excluding patients in AII rooms, there were seven, five, four, and three COVID‐19 positive patients on wards served by the AHUs studied on each of the four respective sampling days. As expected, there was no identifiable relationship between observed viral load and the number of COVID‐19 positive occupants associated with each AHU on the sampling days. In order to utilize AHUs as an environmental surveillance method, aerosol samples could be collected at the AHUs to get a temporally resolved viral load signal. Using filter swabs, a 1–2 week rolling average could potentially integrate enough new data to reveal trends in human occupancy viral load, similar to current wastewater surveillance initiatives.^51^

## 5 CONCLUSION

This study demonstrates SARS‐CoV‐2 RNA contamination throughout several AHUs path of flow, including return air, two filtration stages, and supply air, for multiple floors of the hospital and serves as the first evidence of the potential for SARS‐CoV‐2 RNA (and possibly virus), irrespective of viability, to enter into and travel throughout HVAC systems. While there is still a paucity of information on the potential viability and infectivity of SARS‐CoV‐2, this paper demonstrates that actions to protect against the potential for SARS‐CoV‐2 aerosol travel, and subsequent transmission, should be taken into account in built environment mitigation strategies. Specifically, the data suggest that actions should be taken by healthcare facilities to avoid or minimize potential future SARS‐CoV‐2 healthcare associated infection. Finally, this study suggests the potential utility for AHUs to serve as a common shared air location to conduct environmental viral surveillance that could guide and evaluate the effectiveness of environmental viral transmission mitigation strategies, including building ventilation operation, human behaviors, targeted surveillance, and targeted decontamination, in response to measured viral loads.

## AUTHOR CONTRIBUTIONS

PFH, LGD, MF, and KVDW conceived of the project scope and MF, JMT, RGM, WBM, and KVDW oversaw project and manuscript development. AW provided significant technical knowledge and access to hospital HVAC systems and air handling units. PFH, LD, and DAC performed sample collection, and initial processing. PFH and LD performed sample transport, nucleic acid isolation, and nucleic acid analysis. PFH performed analysis of raw abundance data and contributed to figure and table production. MF and AW developed Figure 1. PFH and LD wrote the initial manuscript and KVDW, MF, JMT, WBM, DAC, RGM, and AW provided significant edits to the manuscript. KVDW and PFH managed peer‐reviewer feedback.

## Supporting information

Figure S1

Table S1

Supplementary Material

Supplementary Material

## Data Availability

The data that supports the findings of this study are available in the [Link], [Link] of this article.
