# Supplementary figures and images for: Identification of SARS‐CoV‐2 RNA in healthcare heating, ventilation, and air conditioning units
Source: Indoor Air. 2021 Jun 29;31(6):1826–32. doi: 10.1111/ina.12898 (PMC8447041; doi:10.1111/ina.12898)

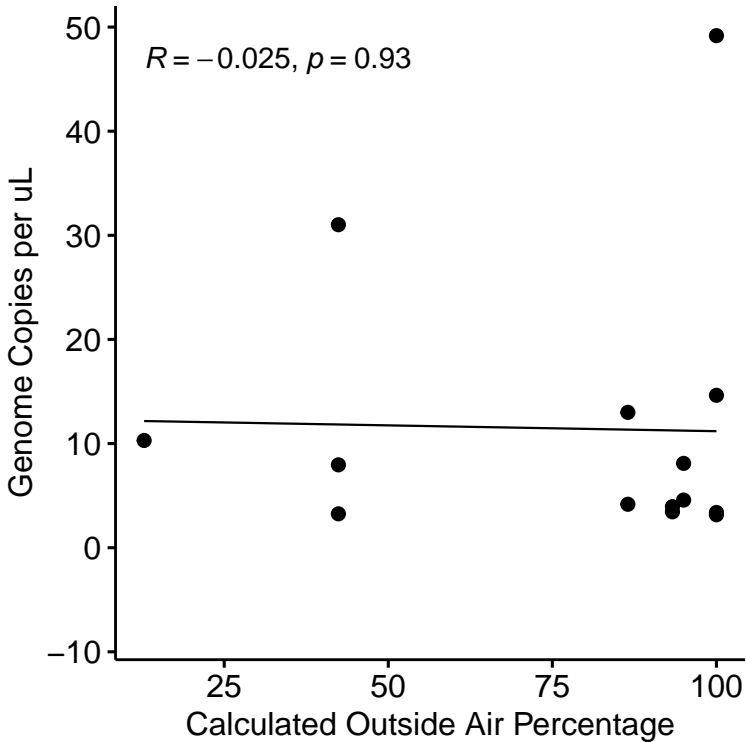

Supplement: Supplementary file 1 — Figure S1 [file INA-31-1826-s002.pdf]
