# Supplementary material for: Identification of SARS‐CoV‐2 RNA in healthcare heating, ventilation, and air conditioning units
Source: Indoor Air. 2021 Jun 29;31(6):1826–32. doi: 10.1111/ina.12898 (PMC8447041; doi:10.1111/ina.12898)
Supplement: Supplementary file 3 — Supplementary Material [file INA-31-1826-s003.docx]

**Filter Change Data**

AHU 1301

Prefilter Change Date - 14 May 2018

Final Filter Change Date - 19 April 2017

AHU 1305

Prefilter (coils) Cleaning Date - 19 May 2020

Final Filter Change Date - 25 March 2020

AHU 1310

Prefilter Change Date - 11 March 2020

Final Filter Change Date - 26 September 2017
