# Supplementary material for: Identification of SARS‐CoV‐2 RNA in healthcare heating, ventilation, and air conditioning units
Source: Indoor Air. 2021 Jun 29;31(6):1826–32. doi: 10.1111/ina.12898 (PMC8447041; doi:10.1111/ina.12898)
Supplement: Supplementary file 4 — Supplementary Material [file INA-31-1826-s001.docx]

**Primers and Artificial Gene Standard**

**SARS-CoV-2 Spike F:**

CCTACTAAATTAAATGATCTCTGCTTTACT

**SARS-CoV-2 Spike R:**

CAAGCTATAACGCAGCCTGTA

**SARS-CoV-2 S Gene Fragment Standard:**

TGTGCCCTTTTGGTGAAGTTTTTAACGCCACCAGATTTGCATCTGTTTATGCTTGGAACAGGAAGAGAATCAGCAACTGTGTTGCTGATTATTCTGTCCTATATAATTCCGCATCATTTTCCACTTTTAAGTGTTATGGAGTGTCTCCTACTAAATTAAATGATCTCTGCTTTACTAATGTCTATGCAGATTCATTTGTAATTAGAGGTGATGAAGTCAGACAAATCGCTCCAGGGCAAACTGGAAGATTGCTGATTATAATTATAAATTACCAGATGATTTTACAGGCTGCGTTATAGCTTGGAATTCTAACAATCTTGATTCTAAGGTTGGTGGTAATTATAATTACCTGTATAGATTGTTTAGGAAGTCTAATCTCAAACCTTTTGAGAGAGATATTTCAACTGAAATCTATCAGGCCGGTAGCACACCTTGTAA
